# EphA2 is a functional entry receptor for HCMV infection of glioblastoma cells

**DOI:** 10.1371/journal.ppat.1011304

**Published:** 2023-05-05

**Authors:** Xiao-Dong Dong, Yan Li, Ying Li, Cong Sun, Shang-Xin Liu, Hao Duan, Run Cui, Qian Zhong, Yong-Gao Mou, Le Wen, Bo Yang, Mu-Sheng Zeng, Min-Hua Luo, Hua Zhang

**Affiliations:** 1 State Key Laboratory of Oncology in South China, Collaborative Innovation Center for Cancer Medicine, Guangdong Key Laboratory of Nasopharyngeal Carcinoma Diagnosis and Therapy, Sun Yat-sen University Cancer Center, Guangzhou, China; 2 Department of Pathology, Sun Yat-sen University Cancer Center, Guangzhou, China; 3 MOE Key Laboratory of Tropical Disease Control, Shenzhen Centre for Infection and Immunity Studies (CIIS), School of Medicine, Shenzhen Campus of Sun Yat-sen University, Shenzhen, Guangdong, China; 4 State Key Laboratory of Virology, CAS Center for Excellence in Brain Science and Intelligence Technology, Wuhan Institute of Virology, Chinese Academy of Sciences, Wuhan, China; 5 The Joint Center of Translational Precision Medicine, Guangzhou Institute of Pediatrics, Guangzhou Women and Children Medical Center; Wuhan Institute of Virology, Chinese Academy of Sciences, China; Leibniz Institute of Virology (LIV), GERMANY

## Abstract

Human cytomegalovirus (HCMV) infection is associated with human glioblastoma, the most common and aggressive primary brain tumor, but the underlying infection mechanism has not been fully demonstrated. Here, we show that EphA2 was upregulated in glioblastoma and correlated with the poor prognosis of the patients. EphA2 silencing inhibits, whereas overexpression promotes HCMV infection, establishing EphA2 as a crucial cell factor for HCMV infection of glioblastoma cells. Mechanistically, EphA2 binds to HCMV gH/gL complex to mediate membrane fusion. Importantly, the HCMV infection was inhibited by the treatment of inhibitor or antibody targeting EphA2 in glioblastoma cells. Furthermore, HCMV infection was also impaired in optimal glioblastoma organoids by EphA2 inhibitor. Taken together, we propose EphA2 as a crucial cell factor for HCMV infection in glioblastoma cells and a potential target for intervention.

## Author summary

Human cytomegalovirus (HCMV) belonged to β-human herpesvirus is a ubiquitous pathogen causing congenital infection and relating to morbidity and mortality in immunocompromised transplant patients. Moreover, HCMV has been demonstrated to promote the progression of glioblastoma, the most common and aggressive primary brain tumor. Many studies highlighted the inevitable relationship between HCMV and glioblastoma. But the underlying mechanism of HCMV infection of glioblastoma cells has not been fully demonstrated. Here we found that EphA2, a member of receptor tyrosine kinases (RTKs) family, played a crucial role in HCMV infection of glioblastoma cells. Our research suggests that EphA2 mediates the infection of HCMV by interacting with glycoproteins gH/gL/gO displaying on the surface of HCMV virion. We find that anti-EphA2 antibody and 2,5-dimethylpyrrolyl benzoic acid derivatives can block HCMV infection in glioblastoma cells, while 2,5-dimethylpyrrolyl benzoic acid derivatives can block HCMV infection in GBOs in a dose-dependent manner, indicating that the strategy of developing an anti-HCMV drug targeting EphA2 is feasible. Our findings may assist future studies striving for a better understanding of how HCMV infects glioblastoma cells and for potential new targets of innovative antiviral strategies.

## Introduction

Glioblastoma, as the most common and aggressive primary brain tumor, exhibits an approximately 15 months median longevity following medical treatment [1]. Many studies highlighted the inevitable relationship between human cytomegalovirus (HCMV) and glioblastoma. The viral proteins immediate-early 1 (IE1), pp65, or HCMV nucleic acids, have been detected in glioblastoma specimens [2–6], and IE1 is negatively associated with patient survival and median longevity [7]. HCMV can promote the progression of glioblastoma through various mechanisms, including affecting the cell cycle, invasion, and metastasis of tumor cells [8–10]. The antiviral drug ganciclovir can restore temozolomide sensitivity, indicating a potential mechanism underlying the positive effects observed in glioblastoma patients treated with antiviral therapy [11]. But the role of HCMV in the pathogenesis of glioblastoma still remains controversial [12].

As a ubiquitous β-human herpesvirus, HCMV is an important pathogen in immunocompromised individuals and tumor patients, causes congenital infection which targets the neural progenitor/stem cells in fetal brain [13]. In vivo HCMV infects and replicates in a wide range of cells, such as epithelial cells, smooth muscle cells, fibroblasts, macrophages, dendritic cells, liver cells, and vascular endothelial cells [14,15]. The widespread cellular tropism promotes the systematic spread of the virus in the human body. The glycoproteins on the surface of the envelope are key molecules that mediate virus infection. There are at least 25 glycoproteins displaying on the surface of HCMV virion, including gB, gM, gO, gH/gL, and UL128-131 [16–18]. Infection of all cell types thus far tested seems to require gH/gL/gO, whereas gH/gL/UL128-131 is an additional requirement some cell types, like epithelial and endothelial cells [19–21]. However, the mechanism of HCMV infection in glioblastoma has remained poorly clear.

Eph receptors are the largest family of receptor tyrosine kinases (RTKs), consisting of 10 EphA receptor members and 5 EphB receptor members. These receptors play important roles in various developmental, physiological, and pathological processes [22]. Specifically, more and more evidence has shown Eph receptors’ involvement in tumorigenesis. The increased expression of ephrin receptor A2 (EphA2) and ephrin receptor A3 (EphA3) correlated with poor prognosis in patients with glioblastomas [23,24]. Additionally, the Eph receptor and its Ephrin ligand were reported to mediate herpesvirus infection. For example, EphA2 and Ephrin receptor A4 (EphA4) were required for Kaposi’s sarcoma-associated herpesvirus (KSHV) infection [25–27]. We and others identified EphA2, which is bound to gH/gL, to serve as a receptor for EBV infection of epithelial cells [28,29]. To explore whether Eph receptors implicate in HCMV infection of glioblastoma, we used siRNA screening to target the members of the Eph receptor family and found that EphA2 played a crucial role in HCMV infection.

## Results

### Eph receptor family siRNA screening identifies EphA2 as a host factor in HCMV infection of glioblastoma cells

The Eph receptors mRNA levels were analyzed using the online database Gene Expression Profiling Interactive Analysis (GEPIA) to determine Eph receptors expression in glioblastoma and normal brain tissues [30]. The results revealed that EphA2, EphB2, EphB3, and EphB4 were significantly upregulated, while EphA4 and EphB6 were significantly downregulated in glioblastoma than normal brain tissues, respectively (S1 and S2 Figs). The mRNA expression of other Eph receptors showed no significant difference between glioblastoma and normal brain tissues (S2 Fig). Furthermore, the prognosis potential of Eph receptors in glioblastoma was determined using GEPIA. Higher EphA2 and EphA8 expression were associated with poorer, while higher EphA3 expression was associated with better, prognosis in patients with glioblastoma, respectively (S1 and S3 Figs). There was no obvious correlation between the expression of other Eph receptors and prognosis (S3 Fig).

**Fig 1:**
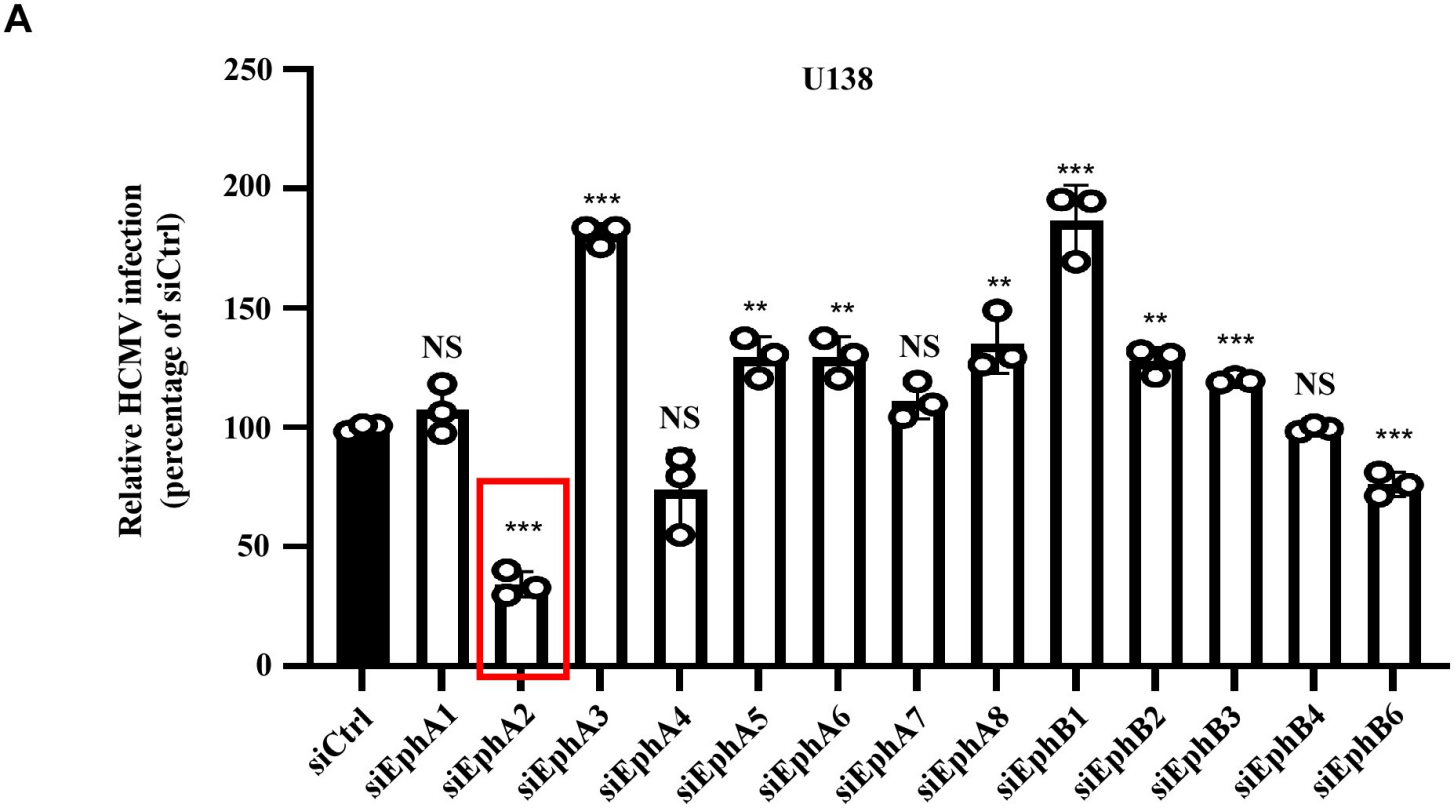
Identifying EphA2 as a potential cellular factor to mediate HCMV infection in glioblastoma cells. **A**, U138 cells were transfected with siRNA pools targeting the Eph receptors family for 36 h. Then cells were infected with HCMV for 3 days, and HCMV-positive cells were quantified by flow cytometry. Bars represent the percentage of infection determined by flow cytometry, with infection of control siRNA duplex (siCtrl) transfected cells normalized to 100%. Data are mean ± s.e.m. (n = 3 biological replicates) and represent three independent experiments. One-way ANOVA was carried out with Dunnett’s correction for multiple comparisons. NS, not significant; **P<0.01; ***P < 0.001.

To explore the potential cell factors associated with HCMV infection in glioblastoma, we performed a siRNA screening in glioblastoma cells U138 targeting the Eph receptors family with a pool of 4 siRNAs targeting each gene. Each siRNA pool was transfected into U138 individually for 48 h, then infected with a reconstructed HCMV Towne strain (ATCC-VR977) expressing GFP [31,32]. The RT-qPCR assay showed that each of these siRNA pools efficiently reduced the expression of its targeted genes (S4 Fig). HCMV-positive cells were determined by flow cytometry, which revealed that knockdown EphA2 and EphB6 reduced the percentage of HCMV infected cells compared to the control siRNA transfected cells (Fig 1A). Notably, EphA2 knockdown resulted a more than 50 percent reduction of HCMV infection (Fig 1A). Therefore, we selected EphA2 for further investigation.

### EphA2 plays a crucial role in HCMV infection of glioblastoma cells

**Fig 2:**
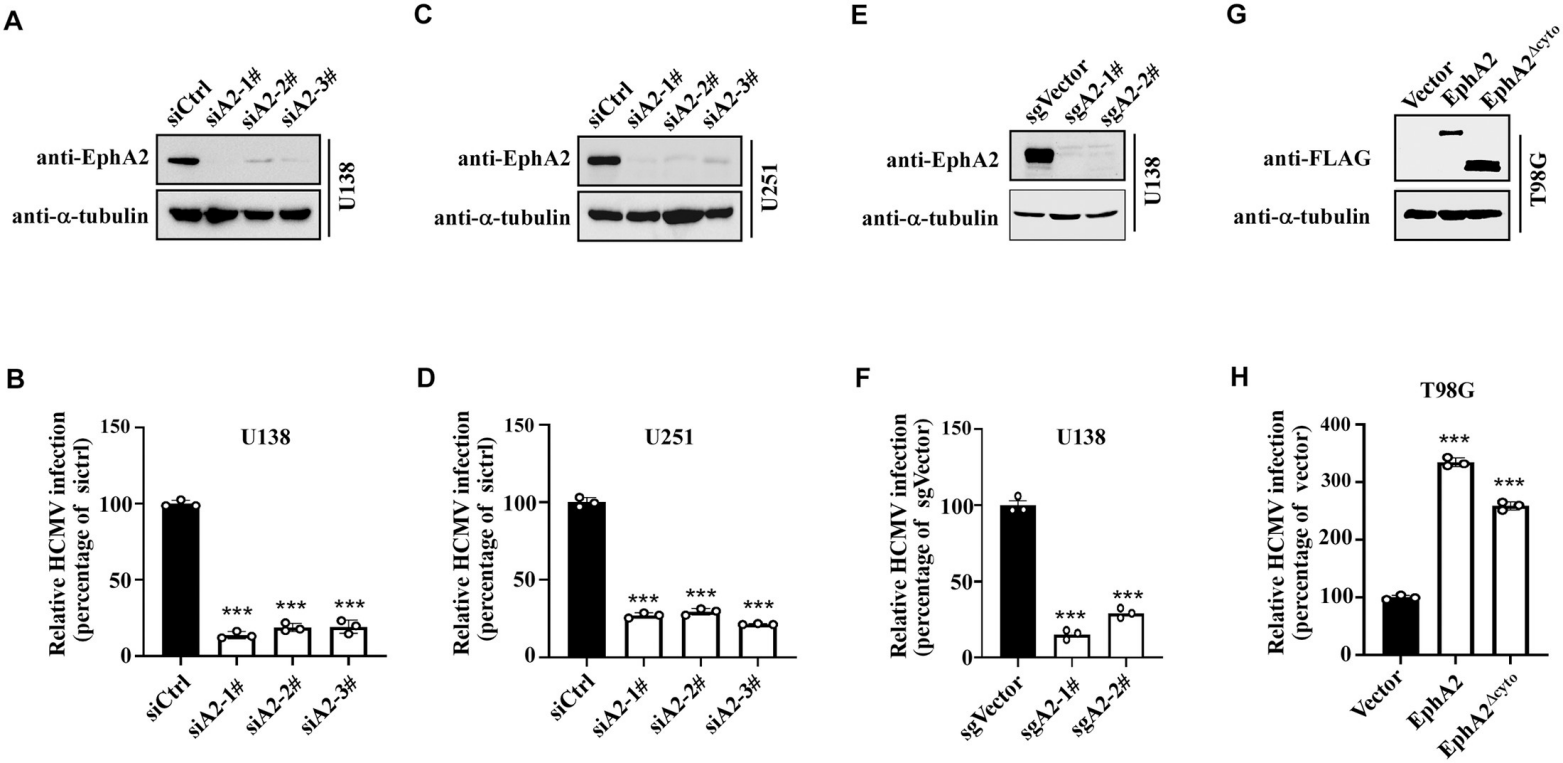
EphA2 plays a key role in HCMV infection of glioblastoma cells. **A to D**, The U138 cells (A) and U251 cells (C) were transfected with EphA2 siRNAs (siA2-1#, siA2-2#, siA2-3#) or siCtrl for 36 h. Part of the cells was harvested, and their EphA2 protein level was analyzed by WB, using α-tubulin as a loading control (representative of 3 independent experiments). The remaining cells were infected with HCMV and HCMV-positive cells and then were analyzed by flow cytometry (B and D). Bars represent the percentage of infection determined by flow cytometry, with infection of siCtrl transfected cells normalized to 100%. Data are mean ± s.e.m. (n = 3 biological replicates) and represent 3 independent experiments. One-way ANOVA was carried out with Dunnett’s correction for multiple comparisons. ***P < 0.001. **E, F**, sgRNAs targeting EphA2 (sgA2-1#, sgA2-2#) were delivered into U138 cells by the lentivirus package system. Cells were selected by puromycin for 3 days. The empty vector was used as control (sgVector). Part of the cells was harvested, and their EphA2 protein level was analyzed by WB (E), using α-tubulin as a loading control (representative of three independent experiments). The remaining cells were infected with HCMV and HCMV-positive cells and then were analyzed by flow cytometry (F). Bars represent the percentage of infection determined by flow cytometry, with infection of sgVector transduced cells normalized to 100%. Data are mean ± s.e.m. (n = 3 biological replicates) and represent 3 independent experiments. One-way ANOVA was carried out with Dunnett’s correction for multiple comparisons. ***P < 0.001. **G, H**, The plasmid expressing Flag-tagged EphA2, EphA2^Δcyto^ or empty vector was stably transduced into T98G cells. Cells were harvested, and the EphA2 expression was analyzed by WB (G). α-tubulin was used as a loading control. Data are representative of 3 independent experiments. HCMV was used to infect these cells, and the HCMV infection efficiency was analyzed by flow cytometry (H). Bars represent the percentage of infection, with infection of empty vector-transfected cells normalized to 1. Data are mean ± s.e.m. (n = 3 biological replicates) and represent 3 independent experiments. One-way ANOVA was carried out with Dunnett’s correction for multiple comparisons. ***P < 0.001.

We further explored the role of EphA2 in HCMV infection in glioblastoma cells. Firstly, we performed EphA2 knockdown in U138 cells by three independent siRNAs. These siRNAs decreased EphA2 protein expression remarkably compared with a siRNA control (Fig 2A). Simultaneously, HCMV infection efficiency was reduced by >70% based on flow cytometry analysis for GFP (Fig 2B). Next, we performed similar experiment on another glioblastoma cell line, U251. Consistently, EphA2 siRNAs also significantly decreased the EphA2 protein expression (Fig 2C) and HCMV infection (Fig 2D). To exclude the potential siRNA off-target effects, we knockout EphA2 using CRISPR/Cas9. EphA2 protein and HCMV infection were remarkably decreased in U138 cells transduced with either sgA2-1# or sgA2-2# sgRNA (Fig 2E and 2F). Altogether, EphA2 is crucial for HCMV infection of glioblastoma cells. As for the reconstructed HCMV Towne strain also can infect fibroblast cells, we also performed the EphA2 knockdown assay in MRC-5 cells. Two independent siRNAs both can reduce the EphA2 expression as well as the HCMV infection efficiency (S5 Fig).

Previous studies showed that deletion of EphA2 intracellular domain (EphA2^Δcyto^) inhibited KSHV infection of both epithelial and endothelial cells [26]. We stably overexpressed the wild-type EphA2 (EphA2^wt^)or EphA2^Δcyto^ in glioblastoma T98G cells (Fig 2G), which were then infected with HCMV. Both EphA2^wt^ and EphA2^Δcyto^ remarkably increased the HCMV infection (Fig 2H). These results demonstrated that the extracellular domain was more essential for HCMV infection of glioblastoma cells, which is consistent with the role of EphA2 in EBV infection of epithelial cells [29].

### EphA2 phisically interacts with HCMV glycoprotein gH/gL

**Fig 3:**
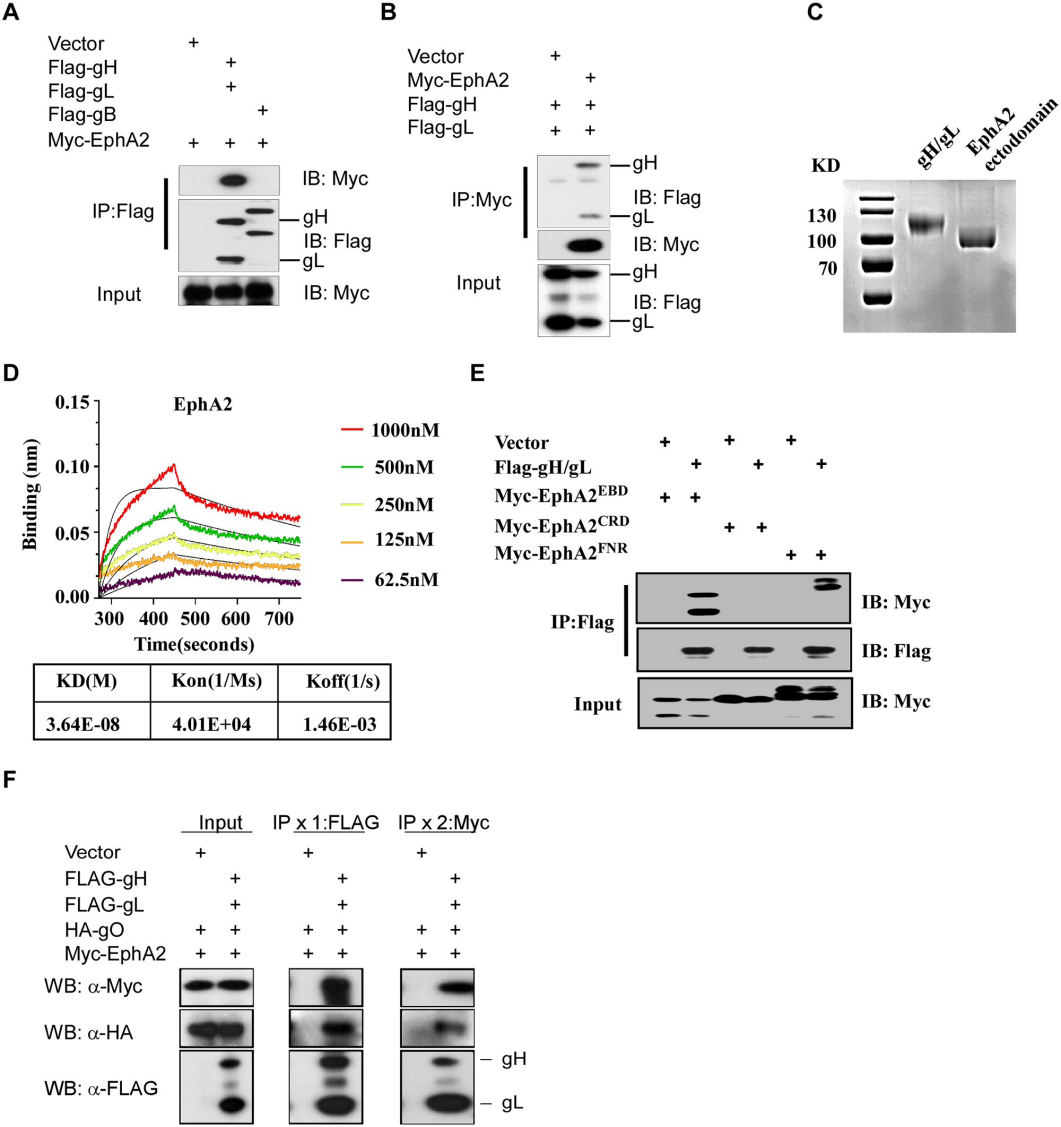
EphA2 interacts with HCMV gH/gL. A, Co-immunoprecipitation assay of Myc tagged EphA2 (Myc-EphA2) with Flag-tagged gH (Flag-gH)/gL(Flag-gL)or Flag-tagged gB (Flag-gB), followed by western blot analysis with indicated antibodies. IB, immunoblotting. Data are representative of 2 independent experiments. B, Co-immunoprecipitation assay of Flag-gH/gL and Myc-EphA2, followed by western blot analysis with indicated antibodies. IB, immunoblotting. Data are representative of 2 independent experiments. C, Purified gH/gL, and EphA2 ectodomain proteins were presented by coomassie blue staining. Data are representative of 2 independent experiments. **D**, BLI analysis for the binding of EphA2 to gH/gL. gH/gL proteins were captured onto SA biosensors and assayed for binding to EphA2 ectodomain proteins at the indicated concentrations. Kinetic values calculated from the fit model for binding curves are shown in the table. E, Co-immunoprecipitation assay of Flag-gH/gL with EphA2 EBD domain (Myc-EphA2^EBD^), EphA2 CRD domain (Myc-EphA2^CRD^) or EphA2 FNR domain (Myc-EphA2^FNR^), followed by western blot analysis with indicated antibodies. IB, immunoblotting. Data are representative of 2 independent experiments. **F**, HEK-293T cells were transfected with Myc-EphA2, HA-gO together with FLAG-gH/gL or vector, lysed, and immunoprecipitated with antibody against FLAG as indicated IPx1:FLAG. The immunoprecipitated proteins were eluted by FLAG peptide and re-immunoprecipitated with antibody against Myc as indicated IPx2:Myc, followed by WB analysis with indicated antibodies. Data are representative of two independent experiments.

The broad tropism of HCMV infection suggested that it interacted with multiple receptors via combination with different glycoproteins to enter different type of host cells [33]. HCMV gH/gL and gB glycoprotein complex was essential for enter host cells [34,35]. Thus, we performed co-immunoprecipitation assays to test if HCMV gH/gL and gB could interact with EphA2 in HEK-293FT cells co-transfected with Myc-tagged EphA2 and Flag-tagged gH/gL or Flag-tagged gB. The results showed that EphA2 was co-immunoprecipitated by Flag-gH/gL pulldown but not Flag-gB (Fig 3A). The interaction of EphA2 with gH/gL was verified by Myc-EphA2 pulldown (Fig 3B).

To further validate whether there was a direct interaction between EphA2 and gH/gL, recombinant His-gH/gL and EphA2 extracellular domains were purified (Fig 3C). Biolayer interferometry (BLI) was used to confirm the direct interaction between EphA2 and gH/gL by demonstrating high-affinity binding of EphA2 extracellular domain to His-gH/gL (KD = 3.64E-08 M) (Fig 3D). Altogether, these results demonstrated a strong, specific, and direct interaction between EphA2 and gH/gL.

The EphA2 ectodomain contains three parts, the Ephrin-binding domain (EBD), the cysteine-rich domain (CRD), and the Fibronectin III type repeats (FNR). We next investigated which EphA2 ectodomain was involved in interaction with gH/gL. So we performed co-immunoprecipitation assays between three EphA2 ectodomains and gH/gL. The result showed that gH/gL interacted with the EphA2 EBD domain and FNR domain but not the CRD domain (Fig 3E).

HCMV gH/gL can form two kinds of complexes Trimer and Pentamer. The HCMV strain we used in this study has defect in genetic integrity and unable to form the Pentamer. We performed the co-IP assay to explore the interaction between EphA2 and the Trimer. The sequential co-IP revealed that both EphA2 and gO were pulled down by anti-Flag immunoprecipitation, followed by anti-Myc immunoprecipitation in cells co-expressing Myc-EphA2, Flag-gH/gL and HA-gO (Fig 3F). These results suggest that EphA2 could also interact with gH/gL/gO trimer.

### EphA2 inhibitor and antibody block HCMV infection of U138 cells in a dose-dependent manner

**Fig 4:**
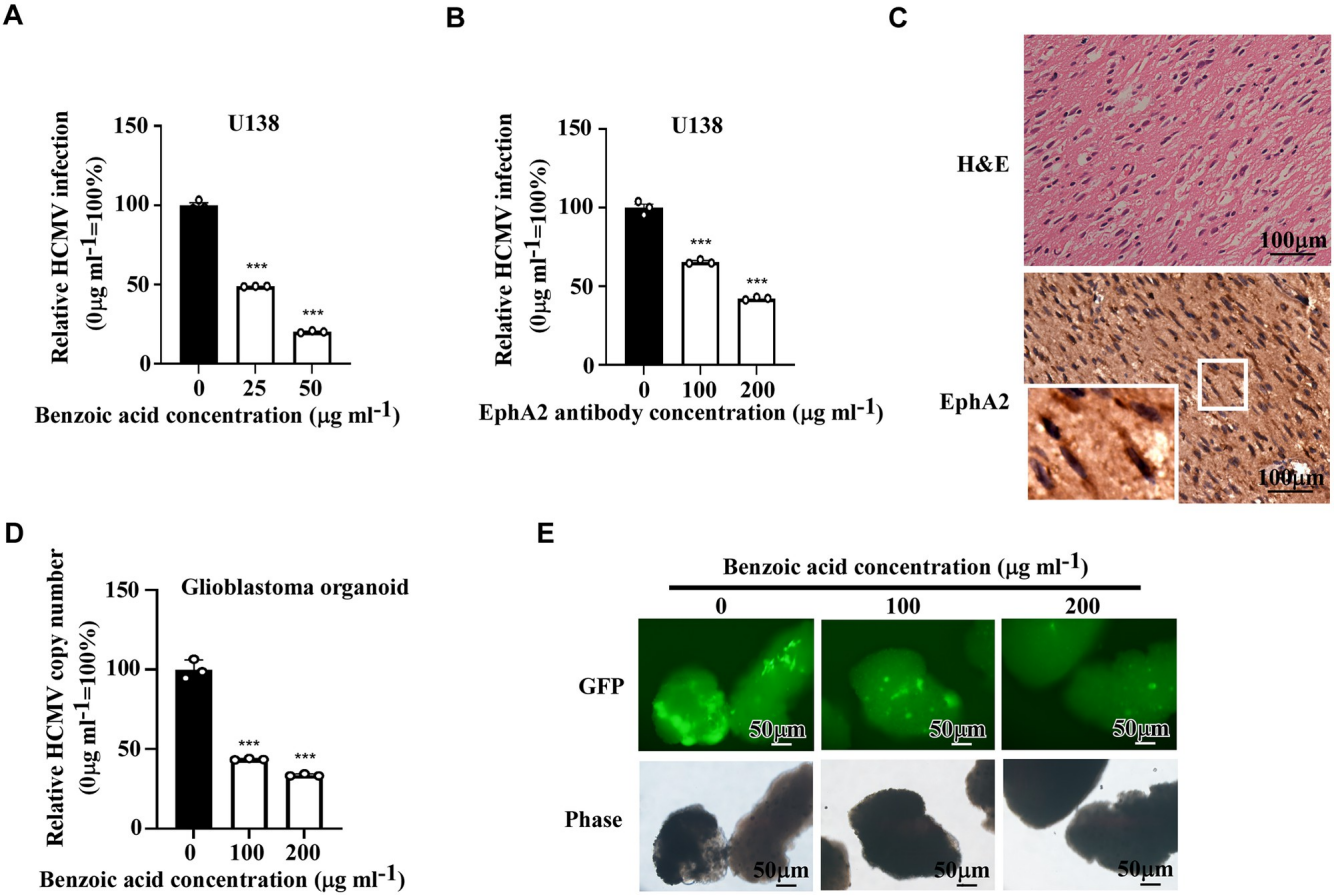
EphA2 inhibitor and anti-EphA2 antibody inhibit HCMV infection in a dose-dependent manner. **A**, U138 cells were infected with HCMV in the presence of an indicated concentration of 2,5-dimethylpyrrolyl benzoic acid derivative. HCMV infection efficiency was quantified by flow cytometry after 3 days. Bars represent the percentage of infection determined by flow cytometry, with infection of no 2,5-dimethylpyrrolyl benzoic acid derivative treated cells normalized to 100%. Data are mean ± s.e.m. (n = 3 biological replicates) and represent 3 independent experiments. One-way ANOVA was carried out with Dunnett’s correction for multiple comparisons. ***P < 0.001. **B**, U138 cells were infected with HCMV in the presence of an indicated concentration of EphA2 antibody or rabbit IgG control. HCMV infection efficiency was quantified by flow cytometry after 3 days. Bars represent the percentage of infection determined by flow cytometry, with infection of rabbit IgG control-treated cells normalized to 100%. Data are mean ± s.e.m. (n = 3 biological replicates) and represent 3 independent experiments. One-way ANOVA was carried out with Dunnett’s correction for multiple comparisons. ***P < 0.001. C, Glioblastoma organoids were stained with hematoxylin-eosin staining (HE) and EphA2 antibody. Images of insets were magnified 3 times. Scale bars: 100 μm. Representative images from the samples were detected. **D, E**, Glioblastoma organoids were infected with HCMV in the presence of an indicated concentration of 2,5-dimethylpyrrolyl benzoic acid derivative. HCMV genome DNA was extracted from HCMV-infected organoids, and the copy number of HCMV was measured using qPCR. The GAPDH DNA was used for cell counting estimation (D). Bars represent relative HCMV DNA copy number determined by qPCR, with the copy number of no 2,5-dimethylpyrrolyl benzoic acid derivative treated cells normalized to 100%. Data are mean ± s.e.m. (n = 3 biological replicates) and represent 2 independent experiments. One-way ANOVA was carried out with Dunnett’s correction for multiple comparisons. ***P < 0.001. HCMV-positive cells were shown green in representative fluorescence images after 3 days (E).

A 2,5-dimethylpyrrolyl benzoic acid derivatives are potent antagonists of EphA2. To evaluate its effects on HCMV infection, U138 cells were first treated with 2,5-dimethylpyrrolyl benzoic acid derivatives and then co-incubated with HCMV. The cytoxic assay showed that 2,5-dimethylpyrrolyl benzoic acid derivatives had no influence on U138 cells proliferation (S6A Fig). The result showed that 2,5-dimethylpyrrolyl benzoic acid derivatives treatment remarkably inhibited HCMV infection of glioblastoma cells in a dose-dependent manner (Fig 4A).

Furthermore, to determine if antibodies against EphA2 ectodomain could block HCMV infection, we generated a rabbit polyclonal antibody against EphA2. Antibody against EphA2 or rabbit IgG control was pre-incubated with U138 cells prior to HCMV infection, and infection efficiency was measured by flow cytometry. Treatment with anti-EphA2 antibody significantly decreased HCMV infection of glioblastoma cells in a dose-dependent manner (Fig 4B) and showed no cytotoxicity (S6B Fig).

Recently, organoids have been applied to model various kinds of tumors. We generated optimal glioblastoma organoids (GBOs) from tissue along the tumor margin with minimal necrosis and little surrounding brain tissue. Immunohistochemistry (IHC) analyses showed that the GBOs resembled their corresponding parental tumors with expression of GFAP and olig-2, high ki-67 proliferation index, and SMA-positive vessel (S7 Fig). Simultaneously, EphA2 could be detected in the GBOs by IHC (Fig 4C).

We then treated the GBOs with 2,5-dimethylpyrrolyl benzoic acid derivatives and then co-incubated with HCMV. Three days post-infection, the qPCR analysis showed that HCMV copy number was reduced in GBOs pretreated with EphA2 inhibitors in a dose-dependent manner (Fig 4D). We also observed under the fluorescence microscope that GBOs were permissive for HCMV infection in vitro, and 2,5-dimethylpyrrolyl benzoic acid derivatives could inhibit HCMV infection of GBOs (Fig 4E). Altogether, these results suggested that blocking EphA2 impairs HCMV infection of glioblastoma cells and highlighted EphA2 as a potential therapeutic target to halt HCMV infection in glioblastoma cells.

### EphA2 mediates HCMV entry and fusion in glioblastoma cells

**Fig 5:**
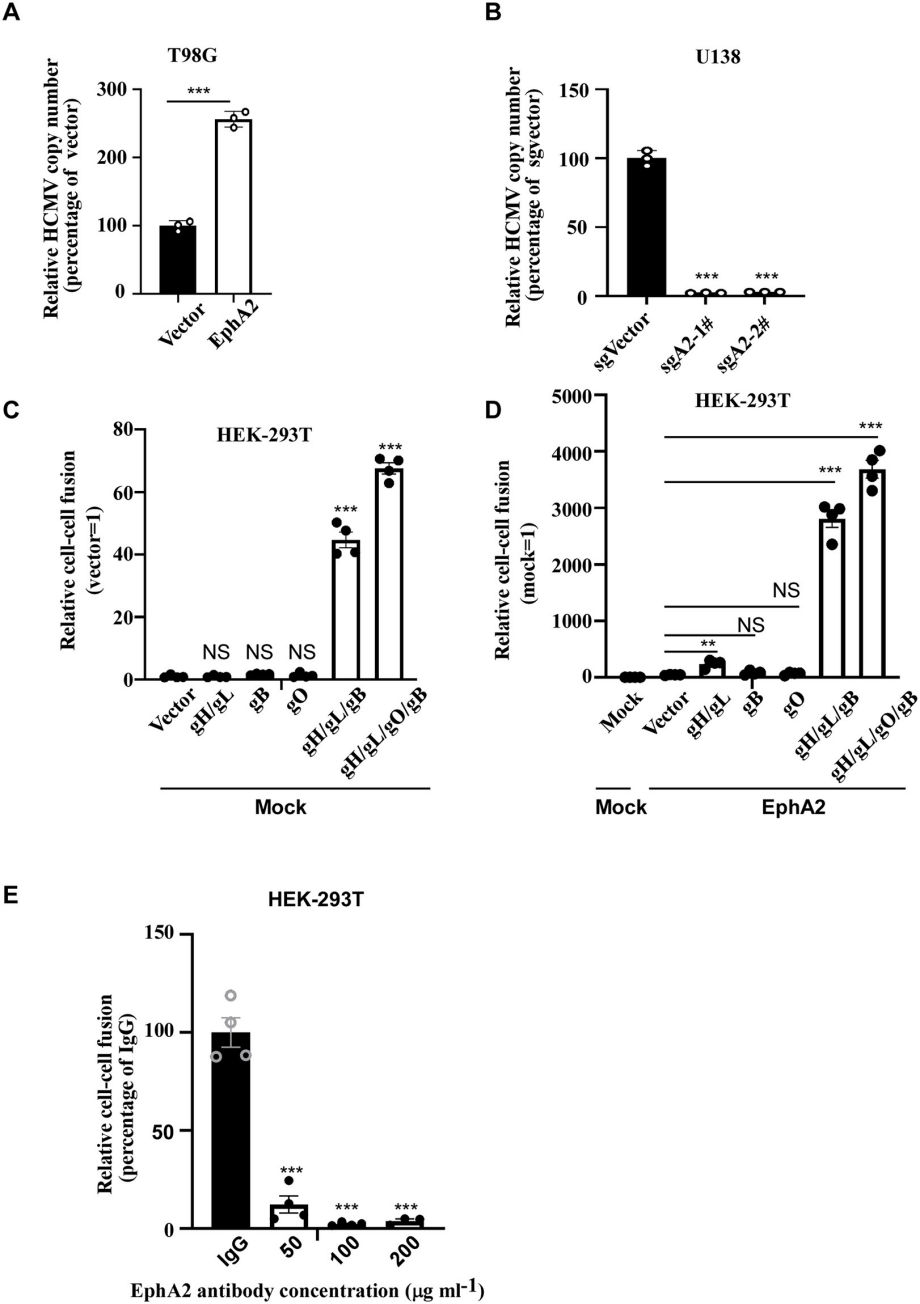
EphA2 mediates HCMV entry and fusion. **A**, The T98G cells stably expressing EphA2 were incubated with HCMV on ice for 2 h and then 30 minutes at 37°C. Cells were washed with Hanks solution three times. The surface-bound virus was removed by proteinase K digestion. Then HCMV DNA copy number per cell was measured by qPCR. Bars represent the percentage of HCMV entry, with the entry of empty vector-transfected cells normalized to 100%. Data are mean ± s.e.m. (n = 3 biological replicates) and represent 2 independent experiments, two-tailed unpaired Student’s *t*-test. ***P < 0.001. **B**, EphA2 knockout cells were incubated with HCMV on ice for 2 h and then 30 minutes at 37°C. Cells were washed with Hanks solution three times. The surface-bound virus was removed by proteinase K digestion. Then HCMV DNA copy number per cell was measured by qPCR. Bars represent the percentage of HCMV entry, with the entry of sgVector transfected cells normalized to 100%. Data are mean ± s.e.m. (n = 3 biological replicates) and represent 2 independent experiments. One-way ANOVA was carried out with Dunnett’scorrection for multiple comparisons. ***P < 0.001. **C**, Effector HEK-293T cells were transfected with pT7-EMCLuc, pRL-SV40 and gB or gH/gL or gO or gH/gL/gB or gH/gL/gO/gB. Cells then were co-cultured with HEK-293T cells transfected with a plasmid for T7 polymerase. The relative fusion activity was calculated as the ratio of firefly to Renilla luciferase activity after 24 h. Data are mean ± s.e.m. (n = 4 biological replicates) and represent 3 independent experiments. One-way ANOVA was carried out with Dunnett’s correction for multiple comparisons. NS *P*>0.05, ***P < 0.001. **D**, Cell-based HCMV fusion assay by co-culturing HEK-293T cells transfected with plasmids expressing EphA2 or vector and HEK-293T transfected with gB or gH/gL or gO or gH/gL/gB or gH/gL/gO/gB. Bars represent the percentage of fusion, with the fusion of vector-transfected cells normalized to 100%. Data are mean ± s.e.m. (n = 4 biological replicates) and represent 2 independent experiments, One-way ANOVA was carried out with Dunnett’s correction for multiple comparisons. NS *P*>0.05, **P < 0.01, ***P < 0.001. **E**, Effector HEK-293T cells were transfected with pT7-EMCLuc, pRL-SV40 and gH/gL/gO/gB. Cells then were co-cultured with HEK-293T cells transfected with a plasmid for T7 polymerase in the presence of an indicated concentration of EphA2 antibody or rabbit IgG control. The relative fusion activity was calculated as the ratio of firefly to Renilla luciferase activity after 24 h. Data are mean ± s.e.m. (n = 4 biological replicates) and represent 3 independent experiments. One-way ANOVA was carried out with Dunnett’s correction for multiple comparisons. ***P < 0.001.

HCMV glycoprotein gB and gM/gN glycoprotein complex were involved in the initial attachment of HCMV particles to host cells [36,37]. The gH/gL complex in concert with gB was believed to mediate the fusion of the virus with cellular membranes [38]. We performed entry assays, which revealed that overexpression of EphA2 in T98G cells promoted HCMV entry (Fig 5A). However, EphA2 knockout inhibited HCMV entry in U138 cells (Fig 5B). We further explored whether EphA2 was required for fusion. Using cell-based HCMV fusion assay, we found that gH/gL or gB alone slightly increased HCMV fusion, while co-expression of gH/gL and gB or gH/gL/gO and gB remarkably enhanced HCMV fusion (Fig 5C). Compared to the vector control, overexpression of EphA2 increased HCMV fusion (Fig 5D). We used HEK-293T cells expressing gH/gL/gO/gB as effector cells and HEK-293T cells as target cells. HEK-293T cells were treated with EphA2 antibody or IgG. EphA2 antibody also can block cell based fusion in a dose-dependent manner (Fig 5E). Altogether, these results indicated that EphA2 promoted the fusion of HCMV and cellular membranes, which was largely depended on the coexistence of gH/gL/gO and gB.

## Discussion

Eph receptor family has been reported to mediate infection of several important human pathogens [25–29]. By siRNA screen of Eph receptors family members, combined with a series of loss-of-function, gain-of-function, and protein-protein interaction assays, we identified EphA2 as an entry co-receptor for HCMV infection of glioblastoma.

The relationship between HCMV and glioblastoma has been debated for a long time [2,5,39,40]. The expression of HCMV proteins in glioblastoma tissues was first reported in 2002. Then, more and more studies have detected HCMV proteins or DNA in human glioblastoma tissue samples [4,41,42]. Moreover, HCMV has been demonstrated to promote the progression of glioblastoma, negatively associate with patient’s survival, and the antiviral drug can restore sensitivity of glioblastoma chemotherapeutic drug [7–11]. These studies supply evidences for a role of HCMV infection in the pathogenesis of glioblastoma and emerging therapeutic interventions directed against HCMV.

Several molecules, such as EGFR, Integrin αvβ3, CD90, CD147, Neuropilin-2, and platelet-derived growth factor receptor alpha (PDGFRα), have been identified as receptors or co-receptors for HCMV infection in fibroblasts, epithelial, or endothelial cells [15,43–46]. However, the receptor(s) that mediates HCMV infection of human glioblastoma cells is unclear. Eph receptors and their Ephrin ligands are strongly related to herpes virus infection. We identified EphA2 as a host factor for HCMV infection of human glioblastoma cells by siRNA screen. EGFR, Integrin αvβ3, Neuropilin-2, and PDGFRα, but not CD90 and CD147, were significantly upregulated in glioblastoma than normal brain tissues by GEPIA analysis (S8 Fig). Whether these genes play some role in the HCMV infection of human glioblastoma cells needs further investigation. It has been reported that HCMV glycoprotein complex gH/gL/gB is important for HCMV fusion [38]. EphA2 also has been found to interact with KSHV gH/gL, EBV gH/gL and gB [25,26,28,29,47]. The binding site of EphA2 on EBV gH/gL (gL NGSN69-72) is different from the binding site of EphA2 on KSHV gH/gL (gH ELEFN50-54) [48]. The three subunits of HCMV Trimer interact in a linear order, where the gL subunits bridge the gH and gO subunits in the center of the complex [49]. In our study, we found that EphA2 interacts with gH N-terminal, and the major binding regions are EBD and FNR domains. It is more like EBV gH/gL but Unlike KSHV gH/gL, for which the EphA2 EBD is the major binding region. The specific binding site of EphA2 and HCMV Trimer need further investigation. We have identified that EphA2 could interact with HCMV gH/gL complex, but not gB to promote cell-cell fusion, which may support the development of gH/gL vaccines or drugs targeting gH/gL to block the HCMV infection. Two gH/gL-containing complexes, gH/gL/gO (trimer) and gH/gL/pUL128-131A (pentamer), regulate viral tropism [15]. Whether EphA2 could interact with gH/gL-containing pentamer need further investigation.

Although many studies have detected HCMV DNA or protein in human glioblastoma tissues, it remains undetermined whether HCMV could directly infect the glioblastoma tissues. Organoids have been applied to model various kinds of tumors and drug screening. We demonstrate that the GBOs model is susceptibility to HCMV infection, suggesting GBOs model could be a potential tool to for studying the role HCMV in glioblastoma as well as for screening anti-HCMV drugs. Furthermore, EphA2 has been found to drive tumorigenicity in glioblastoma [50]. We find that anti-EphA2 antibody and 2,5-dimethylpyrrolyl benzoic acid derivatives can block HCMV infection in glioblastoma cells, while 2,5-dimethylpyrrolyl benzoic acid derivative can block HCMV infection in GBOs in a dose-dependent manner, indicating that the strategy of developing an anti-HCMV drug targeting EphA2 is feasible.

In conclusion, we have identified EphA2 as an important host factor mediating entry and fusion of HCMV with glioblastoma cells, which may assist future studies striving for a better understanding of how HCMV infects glioblastoma cells and for potential new targets of innovative antiviral strategies.

## Materials and methods

### Cell lines

U138, U251, T98G, HFF, MRC-5 and HEK-293T cells (Thermo Scientific, R70007) were grown in DMEM (C11995500BT, GIBCO, California) supplemented with 10% (vol/vol) FBS (10099-141C, GIBCO, Australia). Cells were cultured in humidified 5% CO2 incubators at 37°C. HEK-293T was purchased from ATCC; U251, HFF, MRC-5 were gifted from Professor Luo Minhua (WuHan institute of virology, CAS, China); U138 and T98G were gifted from Professor Chen Zhongping (Sun Yat-sen University, Guangzhou, China).

### Reagents

The reagents used were as follows: antibodies against EphA2 (6997, CST, Massachusetts), α -tubulin (sc-8035, Santa Cruz, Texas), β -actin (66009-1-Ig, Proteintech, Chicago), Myc-tag (16286-1-Ig, Proteintech, Chicago), Flag-tag (66008-1-Ig, Proteintech, Chicago), Flag-tag (F4049, Sigma-Aldrich, Germany), normal rabbit IgG (AB-105-C, R&D, Minnesota), the horseradish peroxidase (HRP)-conjugated goat-anti-mouse/rabbit secondary antibodies (#31460, #61–6520, Invitrogen, California), IgG (AB-105-c, R&D, Minnesota) and 2,5-dimethylpyrrolyl benzoic acid derivative (sc-314230, Santa Cruz, Texas). All other reagents were obtained from Sigma-Aldrich unless otherwise indicated.

### Gene silencing assays

The siRNAs pools against Eph family genes and non-targeting siRNA duplexes denoted as siCtrl were synthesized from RIBOBIO (China). The three single siRNA duplexes against EphA2 (RIBOBIO, China) were listed as follows: EphA2 si1#: 5ʹ-GCAGCAAGGTGCACGAATT-3ʹ; EphA2 si2#: 5ʹ-TGACCAACGACGACATCAA-3ʹ; EphA2 si3#: 5ʹ-GCAGACTGTGAACTTGACT-3ʹ. Under the instructions, all the siRNAs were delivered by RNAi MAX (13778150; Invitrogen, California).

### RT-qPCR

Total RNA was extracted using TRIzol reagent (T9424; Sigma-Aldrich, Germany). 1 μg of RNA was reversely transcribed using the RNA Reverse Transcription System (A5001, Promega, Wisconsin) to analyze gene expression. The mRNA level was calculated by RT-qPCR using the LightCycler 480 SYBR Green I Master (04887352001, Roche, Switzerland) and analyzed on a Roche LightCycler 480. All the gene expressions were normalized to the housekeeping gene actin beta (ACTB).

### Plasmids

For HCMV infection of T98G assays, cDNA of EphA2 and EphA2^Δcyto^ were integrated into pHAGE vector; for co-IP assays, cDNA of EphA2, EphA2^ΔEBD^, EphA2^ΔCRD^, EphA2^ΔFNR^, gB, gH, gL were integrated into the pCDNA6-Myc vector, Flag-tagged gH, gL, were integrated into pCDNA3.1 vector; for cell-based fusion assay, expression plasmids for pCAG-T7, pT7EMC-Luc were gifted from Professor R. Longnecker (Northwestern University), and Wolfgang Hammerschmidt (Helmholtz Zentrum München), cDNA of gH, gL, gB were integrated into Phage vector; for purification assay, cDNA of EphA2 (27–534) was integrated into pCDNA3.1 vector with N-terminal Kozak sequence and CD5 signal peptide and C-terminal 6*Histidine tag, pLko.-gH-gL was gifted from Professor Qian Zhikang (Fudan University).

### Plasmid transfection

Indicated plasmids were delivered by Lipofectamine 3000 followed the instructions. The pHAGE-EphA2 or pHAGE- EphA2^Δcyto^ was delivered into T98G cells by a lentivirus package system.

### CRISPR-Cas9-mediated EphA2 gene knockout

The EphA2 U138 knockout cell line was generated by CRISPR-Cas9 gene-editing technology. The two guide RNA (sgRNA) sequences are gRNA-1 5ʹ-GAAGCGCGGCATGGAGCTCC-3ʹ and gRNA-2 5ʹ-CGAAGCAGGCGCGGGCTGCC-3ʹ. Oligonucleotides corresponding to the sgRNA were synthesized and cloned into Cas9-expressing plasmid lenti-CRISPR-V2. EphA2 gRNA-encoding plasmids were packaged into lentivirus and infected U138 cells to establish stable cells. Cells were screened by puro (1 μg mL^-1^) for 3 days.

### HCMV preparation and infection of cells

Recombinant HCMV virus (rHCMV), derived from Towne strain by inserting a GFP gene driven by an SV40 promoter into the viral genome [51]. To provide flanking DNA for homologous recombination HCMV BAC, two fragments of HCMV DNA were PCR amplified from cosmid clone CM1052, which contains the HindIII K/Q, X, V, and Wfragments of AD169 HCMV. This final construct, pUSF-3, contains the prokaryotic genetic elements necessary to confer maintenance as a BAC in E. coli, HCMV DNA sequences to direct homologous recombination to the unique short (US) region of the viral genome, and the GFP marker to facilitate identification and purification of recombinant HCMV in eukaryotic cells. The flanking DNA deletes 8.9 kb of DNA within the US region of HCMV that has been defined as dispensable for HCMV replication in cell culture, truncating IRS1 after amino acid 719 and removing reading frames US1 to US11 plus the carboxyterminal third of US12. Recombinant virus having pUSF-3 substituted for US1-12 was enriched by plaque purification using the GFP marker. The virus was propagated and titrated in HFFs. The indicated cells were infected with with HCMV and incubated for 3 h at 37°C to allow virus enter into cells, then unbound virus was discarded by washing with PBS three times. Then, cells were cultured in a fresh medium for 72 h, then analyzed GFP positive cells with flow cytometry (cytoFLEX; Beckman).

### HCMV entry assay

5 × 10^4^ cells were seeded in 24-well plates overnight. Cells were incubated with HCMV for 2 h at 4°C to allow virus attach to cells, then moved into incubators at 37°C for 30 min. To remove the unbound HCMV Hanks solution was used to wash cells for three times. Then 200 μl trypsin with EDTA and proteinase K were added for 15 min at 4°C and stopped by a trypsin inhibitor. HCMV genome DNA was extracted from HCMV-infected cells using Omega tissue DNA Mini Kit (D3396, Omega) followed by the manufacturer’s instruction. The copy number of internalized HCMV was measured using TaqMan qPCR. qPCR for the GAPDH DNA was used for cell counting estimation. Primers included 5’-GACTAGTGTGATGCTGGCCAAG-3’ and 5’-GCTACAATAGCCTCTTCCTCATCTG-3’ for HCMV, and 5’-CCCCACACACATGCACTTACC-3’ and 5’- CCTAGTCCCAGGGCTTTGATT-3’ for GAPDH.

### Cell-based fusion assay

Effector HEK-293T cells were transiently transfected with plasmid pT7EMCLuc and pRL-SV40; expressing the luciferase gene driven by the T7 polymerase, Renilla luciferase as internal control. Effector cells were also transiently transfected gH/gL, gO, gB Separately or simultaneously. Target cells (HEK-293T) were transfected with expression plasmid for pCAGT7 (expression T7 DNA polymerase) together with EphA2 or empty vector. At 24 h post transfection, 2.5× 10^5^ effector HEK-293T cells were co-cultured with 2.5×10^5^ target HEK-293T cells in 24-well plates for 24 h. The luciferase activity was measured according to a dual-luciferase reporter assay system (E2920, Promega, Wisconsin) by the GloMax-96 Microplate Luminometer. The ratio of firefly luciferase activity to Renilla luciferase activity was used as the relative fusion activity.

### Blocking assay

For the antibody blocking assays, U138 cells were preincubated with rabbit polyclonal anti-EphA2 antibody costumed from Abmart (China), which were validated by ELISA and western blot, by diluting to the concentrations of 100 and 200 μg ml^–1^ in FBS-free DMEM at 4°C for 1 h. Rabbit IgG at the indicated concentration was used as the negative control. Preincubated cells were exposed to HCMV in the presence of indicated antibodies at 37°C for 3 h. The percentage of HCMV infected cells were determined by flow cytometry at 72 h post-infection. For the EphA2 inhibitor 2,5-dimethylpyrrolyl benzoic acid derivative blocking assays, U138 cells were preincubated with 2,5-dimethylpyrrolyl benzoic acid derivative at the concentrations of 25 and 50 μg ml^–1^ in FBS-free DMEM at room temperature for 1 h. DMSO was used as a control. The pretreated cells were infected with HCMV, and HCMV infection efficiency was determined by flow cytometry at 72 h post-infection.

### Immunoprecipitation

The transfected cells were lysed in lysis buffer: 50 mM Tris-HCl, pH 7.4; 250 mM NaCl; 5 mM EDTA, pH 8.0; 0.5% Nonidet P40 (NP40) containing 1 mM phenylmethylsulfonyl fluoride and Roche Complete protease inhibitor cocktail (04693159001, Roche, Switzerland). After centrifuging at 15,000g for 20 min at 4°C, the supernatant was incubated with 25 μl Anti-c-Myc Agarose Affinity Gel (A7470, Sigma-Aldrich, Germany) or ANTI-FLAG M2 Affinity Gel (A2220, Sigma-Aldrich, Germany) for 2 h at 4°C. After washing three times with lysis buffer to remove unbound proteins, the sample was suspended in 2x SDS-sample buffer and boiled for 10min at 98°C. The complex was then analyzed by western blotting with the indicated antibodies.

### 3D organoid culture

The tumor tissues were obtained from patients medically required in Sun Yat-sen University Cancer Center. Tissues were minced into approximately 0.5 to 1 mm diameter pieces and distributed in ultra-low attachment 6-well culture plates with 4 mL of GBO medium containing 50% DMEM:F12 (Thermo Fisher Scientific, Massachusetts), 50% Neurobasal (Thermo Fisher Scientific, Massachusetts), 1X GlutaMax (Thermo Fisher Scientific, Massachusetts), 1X NEAAs (Thermo Fisher Scientific, Massachusetts), 1X PenStrep (Thermo Fisher Scientific, Massachusetts), 1X N2 supplement (Thermo Fisher Scientific, Massachusetts), 1XB27 w/o vitamin A supplement (Thermo Fisher Scientific, Massachusetts), 1X 2-mercaptoethanol (Thermo Fisher Scientific, Massachusetts), and 2.5 μg/ml human insulin (Sigma-Aldrich, Germany) per well and placed on an orbital shaker rotating at 120 rpm within a 37°C, 5% CO_2_, and 90% humidity sterile incubator. Roughly 75% of the medium was changed every 48 h by tilting the plates at a 45° angle and aspirating the medium above the sunken organoids.

### Immunohistochemistry staining

To detect EphA2, CD68, GFAP, SMA, ki-67 and olig-2 in GBOs, antibodies against EphA2 (ab5386, Abcam, UK), CD68(ZM-0060, ZSGB-Bio, China), GFAP (ZA-0529, ZSGB-Bio, China), SMA (ZM-0003, ZSGB-Bio, China), ki-67(TA800648, ZSGB-Bio, China) and olig-2(ZA-0561, ZSGB-Bio, China) were used as primary antibodies overnight at 4°C. After washing three times in PBST, the tissue sections were incubated with anti-rabbit secondary antibody (1:1000, Zymed, California) or anti-mouse secondary antibody (1:1000, Zymed, California), and then treated with 3-diaminobenzidine tetrahydrochloride for 10 seconds, finally stained with 10% Mayer’s hematoxylin (ZSGB-Bio, China).

### Protein purification

Eukaryotic expression system was used to purify human EphA2 and HCMV gH/gL. Briefly, expression plasmid was mixed with Polyethyleneimine Linear (PEI) MW40000 (40816ES, Yeasen, China) at a molar ratio of 1:3 in 293F medium (UP1000, Union, China), and the mixture was transfected to Expi293F (A14527, ThermoFisher, Massachusetts). Then the cells were cultured at 37°C with 5% CO2 and 120 rpm shaking for 6 days, and the supernatant was harvested by centrifuge, filtered with 0.45 um filtering membrane, and applied to gravity column loaded with Ni Sepharose Excel (17371202, Cytiva, Washington, DC). The column was washed by PBS with 30 mM imidazole and eluted by PBS with 500 mM imidazole. The elution was concentrated by ultracentrifuge and further purified by size exclusion chromatography (SEC) using Superdex 200 increase 10/300GL (28990944, Cytiva, Washington, DC) on an AKTA Pure25M (GE healthcare, Massachusetts).

### Biolayer interferometry

The kinetic assay was performed on Octet Red96 (18–1127, ForteBio, California) to determine the affinity of EphA2 with HCMV gH/gL. Briefly, the HCMV gH/gL was first biotinylated using a sulfo-LC-LC-biotin kit (21335, ThermoFisher, Massachusetts) following the manufacturer’s recommended procedures. During the kinetic assay, PBS with 0.1% Tween20 was used as a kinetic buffer (KB) to dilute protein or equilibration biosensors. To detect the affinity, HCMV gH/gL was loaded on SA biosensors (Fortebio, California) at a concentration of 5 ug/mL. After the baseline process, a series of diluted EphA2 proteins were associated with the biosensors, and the dissociation process followed. During the data processing in Octet data analysis software, the curve (0 mM EphA2) was used as blank to recalibrate the other raw curves, and a global fitting model was used to calculate the general kinetic parameters.

### Statistical analyses

Results are expressed as mean ± s.e.m. from three independent experiments. The unpaired parametric two-sided Student’s t-test was used for statistical analysis involving two-group comparisons, and One-way ANOVA was carried out with Dunnett’s correction for multiple comparisons involving more than two groups (*P< 0.05, **P< 0.01, ***P< 0.001). Statistical analyses were performed with Graphpad Prism (GraphPad Software, San Diego, CA, USA).

## Supporting information

S1 Fig The correlation of EphA2 with glioblastoma patients’ survival. **A**, Analysis of EphA2 expression in glioblastoma tissues and normal brain tissues using the samples in the GEPIA database. **B**, Kaplan Meier survival analysis of overall survival rates between high EphA2 expression group and low expression group in the GEPIA database.
(TIF)

S2 Fig The expression of Eph receptors in glioblastoma compared to normal brain tissues. Analysis of EphA1, EphA3, EphA4, EphA5, EphA6, EphA7, EphA8, EphB1, EphB2, EphB3, EphB4 or EphB6 expression levels in glioblastoma tissues and normal brain tissues using GEPIA database.
(TIF)

S3 Fig The correlation of Eph receptors with glioblastoma patients’ survival. Kaplan Meier survival analysis of overall survival rates between high EphA1, EphA3, EphA4, EphA5, EphA6, EphA7, EphA8, EphB1, EphB2, EphB3, EphB4 or EphB6 expression group and low expression group using GEPIA database.
(TIF)

S4 Fig The knockdown efficiency of siRNA pools. The U138 cells were transfected with siRNA pools targeting the indicated genes or control siRNA (siCtrl) for 36 h. RT-qPCR was used to quantify the mRNA level of the respective targeted gene. Results were quantified relative to the housekeeping gene beta-actin (ACTB) expression and shown as fold-change of mRNA abundance normalized to siCtrl, which was normalized to 100%. Data are mean ± s.e.m. (n = 3 biological replicates) and represent 2 independent experiments, two-tailed unpaired Student’s *t*-test.
(TIF)

S5 Fig EphA2 plays a key role in HCMV infection of fibroblast cells. **A**, The MRC-5 cells were transfected with EphA2 siRNAs (siA2-1#, siA2-2#) or siCtrl for 36 h. Part of the cells was harvested, and their EphA2 protein level was analyzed by WB, using α-tubulin as a loading control (representative of 3 independent experiments). The remaining cells were infected with HCMV and HCMV-positive cells were analyzed by flow cytometry (B). Bars represent the percentage of infection determined by flow cytometry, with infection of siCtrl transfected cells normalized to 100%. Data are mean ± s.e.m. (n = 3 biological replicates) and represent 3 independent experiments. One-way ANOVA was carried out with Dunnett’s correction for multiple comparisons. ***P < 0.001.
(TIF)

S6 Fig Cytotoxic assays of 2,5-dimethylpyrrolyl benzoic acid derivatives and EphA2 antibody. **A, B** MTT assay of U138 cell line treated with benzoic acid at concentration of 0, 25, 50 μg /mL (A) or IgG at concentration of 200 μg /mL or EphA2 antibody at concentration of 100, 200 μg /mL. n = 4 biological replicates.
(TIF)

S7 Fig Verification of the glioblastoma organoids by IHC. Glioblastoma organoids were stained with GFAP, olig-2, ki-67, and SMA antibodies. Images of insets were magnified 3 times. Scale bars: 100 μm. Representative images from the samples were detected.
(TIF)

S8 Fig The expression of identified HCMV receptors or co-receptors in glioblastoma compared to normal brain tissues. EGFR, Integrin αvβ3, Neuropilin-2, PDGFRα, CD90 or CD147 expression levels were analyzed in glioblastoma tissues and normal brain tissues using the GEPIA database.
(TIF)
