# Supplementary figures and images for: EphA2 is a functional entry receptor for HCMV infection of glioblastoma cells
Source: PLoS Pathog. 2023 May 5;19(5):e1011304. doi: 10.1371/journal.ppat.1011304 (PMC10191332; doi:10.1371/journal.ppat.1011304)

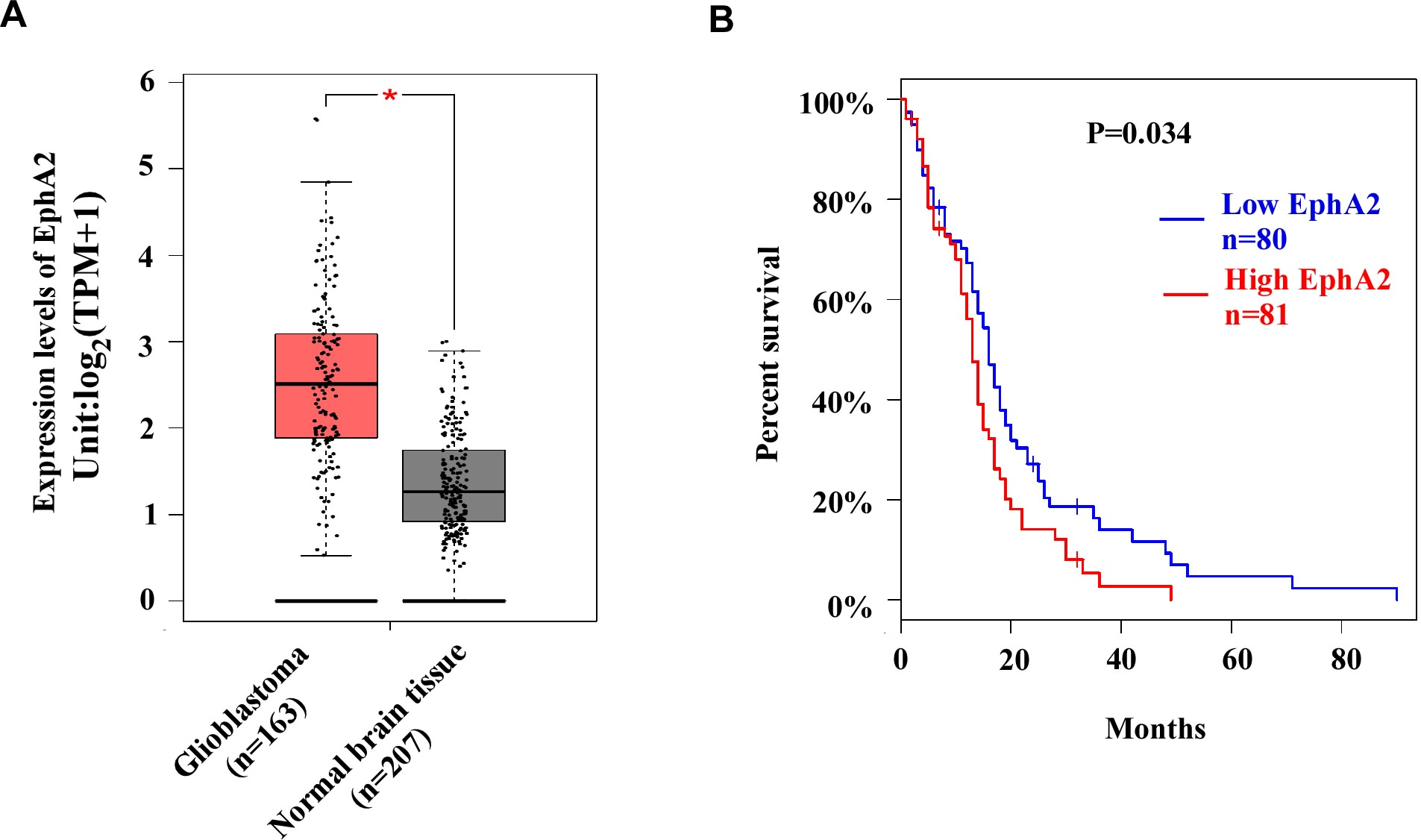

Supplement: S1 Fig — A, Analysis of EphA2 expression in glioblastoma tissues and normal brain tissues using the samples in the GEPIA database. B, Kaplan Meier survival analysis of overall survival rates between high EphA2 expression group and low expression group in the GEPIA database. (TIF) [file ppat.1011304.s001.tif]

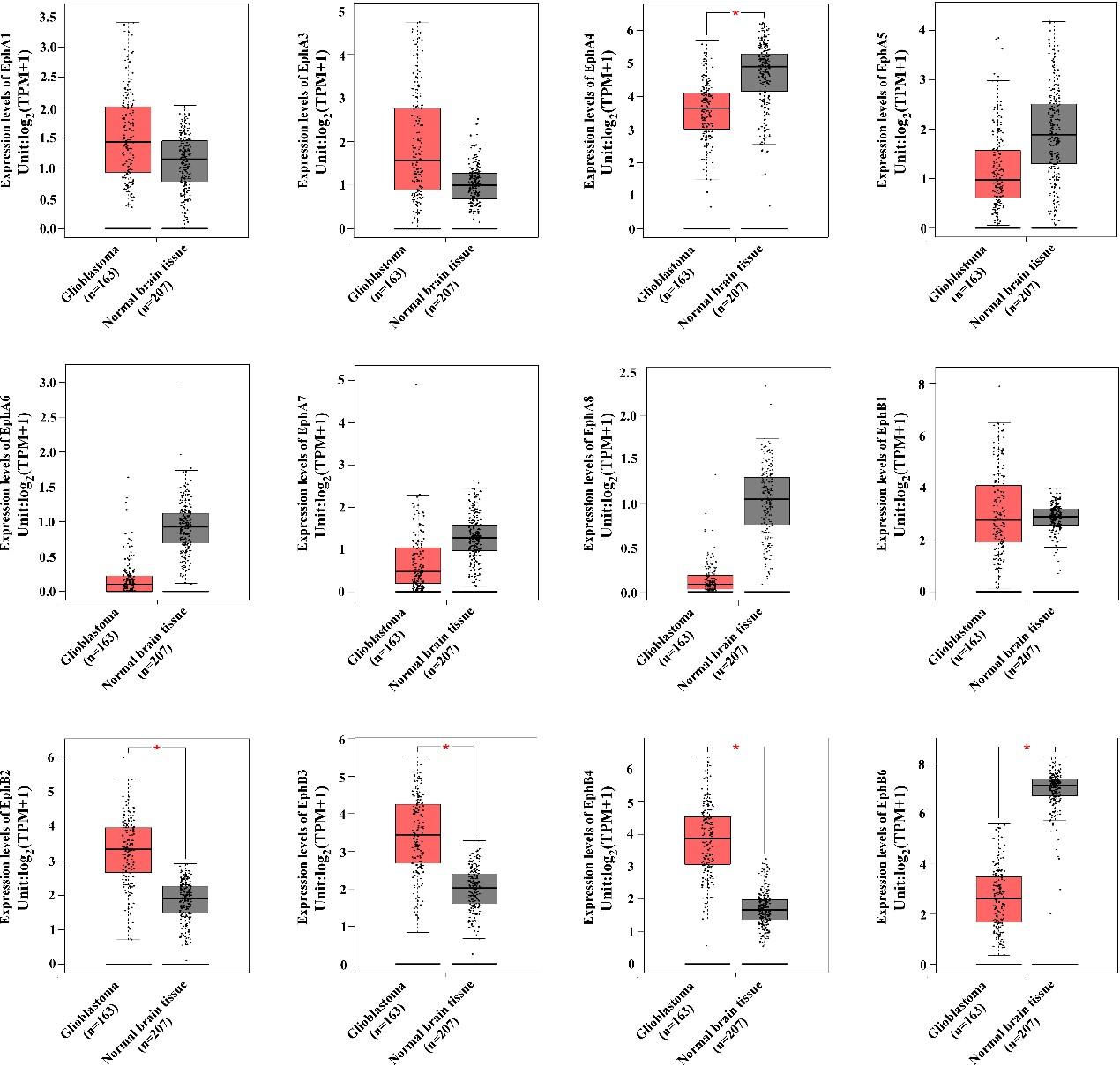

Supplement: S2 Fig — Analysis of EphA1, EphA3, EphA4, EphA5, EphA6, EphA7, EphA8, EphB1, EphB2, EphB3, EphB4 or EphB6 expression levels in glioblastoma tissues and normal brain tissues using GEPIA database. (TIF) [file ppat.1011304.s002.tif]

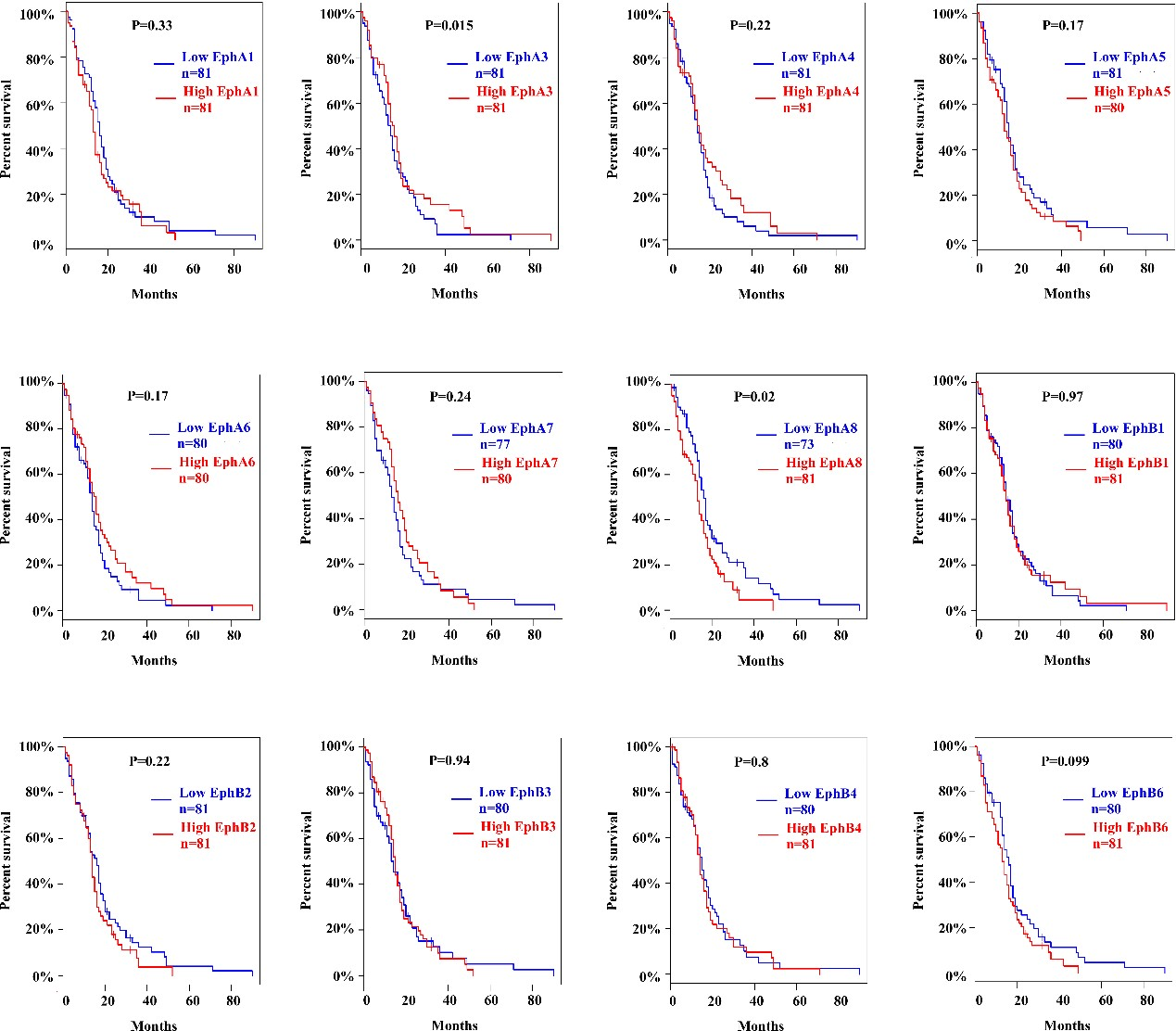

Supplement: S3 Fig — Kaplan Meier survival analysis of overall survival rates between high EphA1, EphA3, EphA4, EphA5, EphA6, EphA7, EphA8, EphB1, EphB2, EphB3, EphB4 or EphB6 expression group and low expression group using GEPIA database. (TIF) [file ppat.1011304.s003.tif]

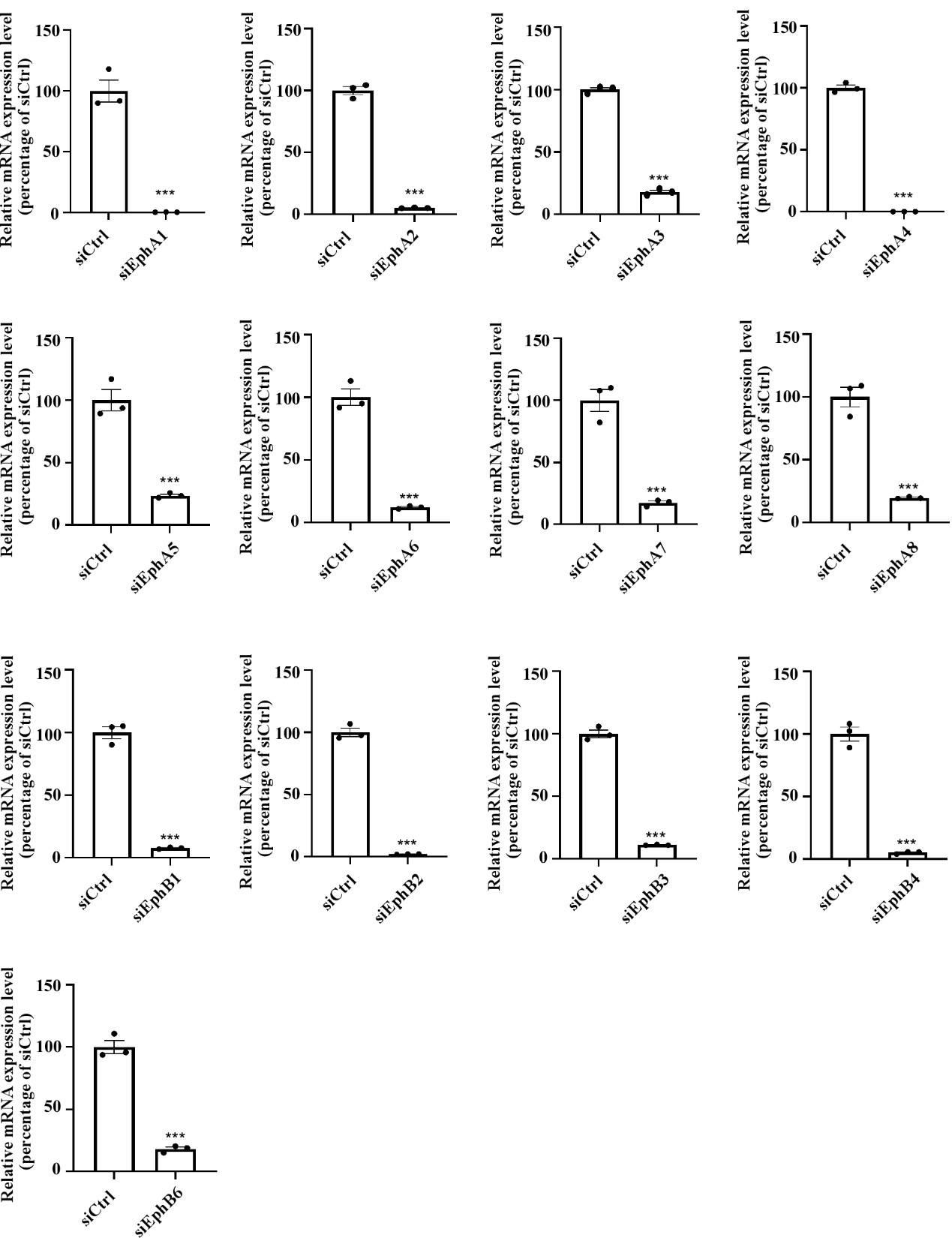

Supplement: S4 Fig — The U138 cells were transfected with siRNA pools targeting the indicated genes or control siRNA (siCtrl) for 36 h. RT-qPCR was used to quantify the mRNA level of the respective targeted gene. Results were quantified relative to the housekeeping gene beta-actin (ACTB) expression and shown as fold-change of mRNA abundance normalized to siCtrl, which was normalized to 100%. Data are mean ± s.e.m. (n = 3 biological replicates) and represent 2 independent experiments, two-tailed unpaired Student’s t-test. (TIF) [file ppat.1011304.s004.tif]

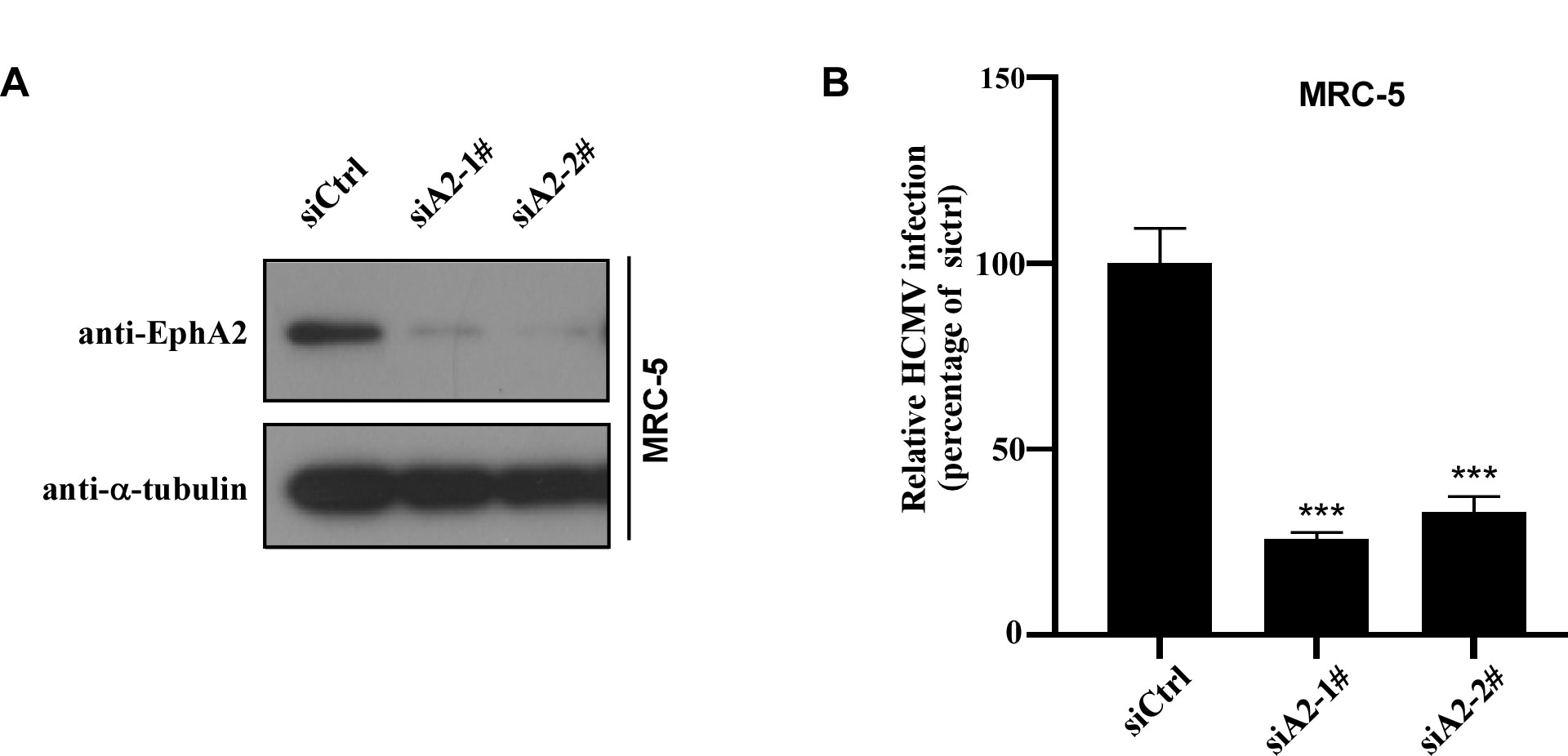

Supplement: S5 Fig — A, The MRC-5 cells were transfected with EphA2 siRNAs (siA2-1#, siA2-2#) or siCtrl for 36 h. Part of the cells was harvested, and their EphA2 protein level was analyzed by WB, using α-tubulin as a loading control (representative of 3 independent experiments). The remaining cells were infected with HCMV and HCMV-positive cells were analyzed by flow cytometry (B). Bars represent the percentage of infection determined by flow cytometry, with infection of siCtrl transfected cells normalized to 100%. Data are mean ± s.e.m. (n = 3 biological replicates) and represent 3 independent experiments. One-way ANOVA was carried out with Dunnett’s correction for multiple comparisons. ***P < 0.001. (TIF) [file ppat.1011304.s005.tif]

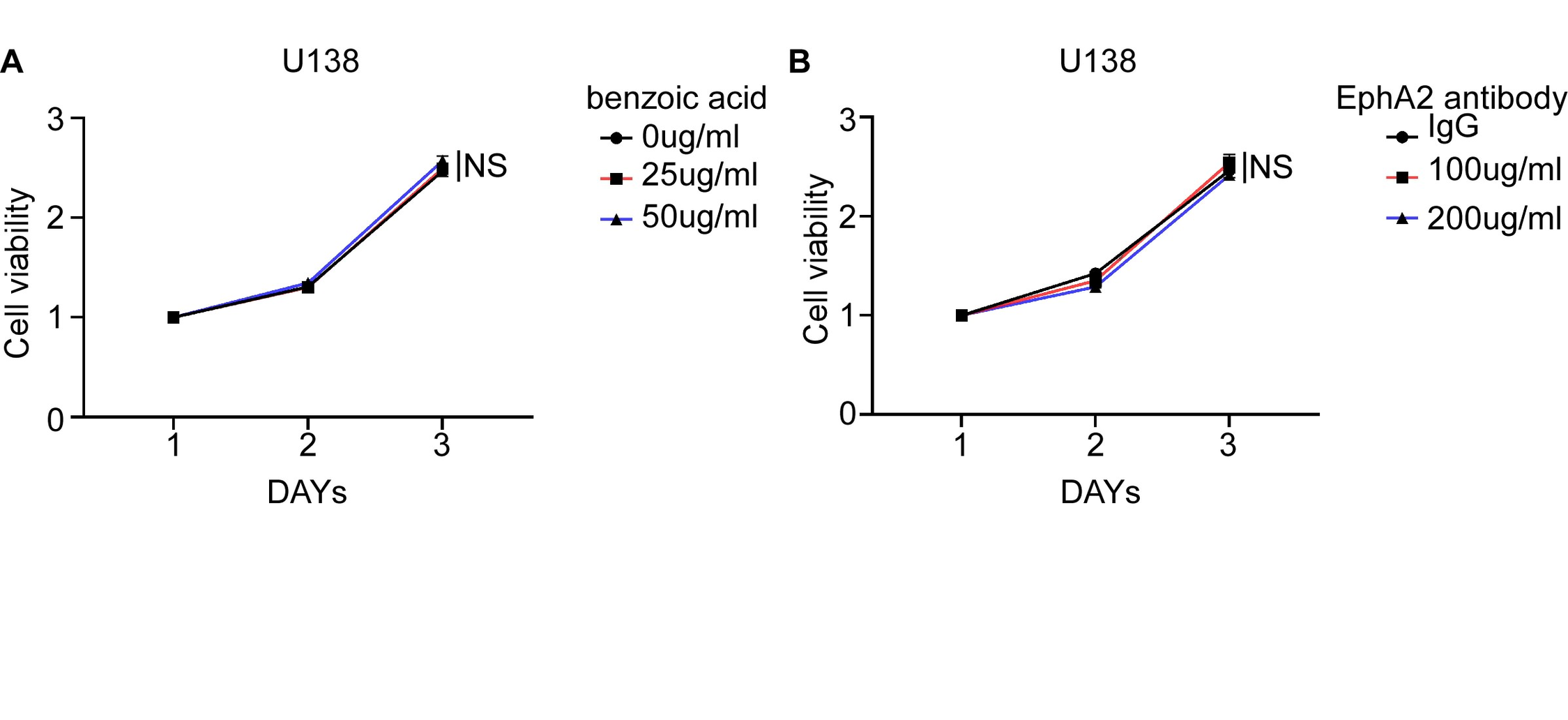

Supplement: S6 Fig — A, B MTT assay of U138 cell line treated with benzoic acid at concentration of 0, 25, 50 μg /mL (A) or IgG at concentration of 200 μg /mL or EphA2 antibody at concentration of 100, 200 μg /mL. n = 4 biological replicates. (TIF) [file ppat.1011304.s006.tif]

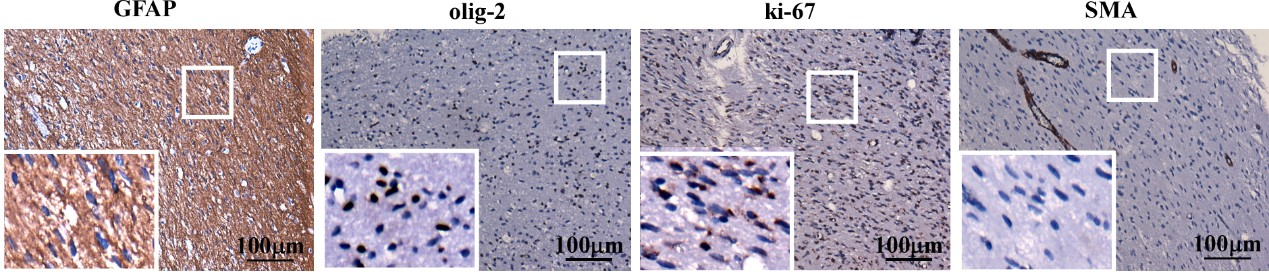

Supplement: S7 Fig — Glioblastoma organoids were stained with GFAP, olig-2, ki-67, and SMA antibodies. Images of insets were magnified 3 times. Scale bars: 100 μm. Representative images from the samples were detected. (TIF) [file ppat.1011304.s007.tif]

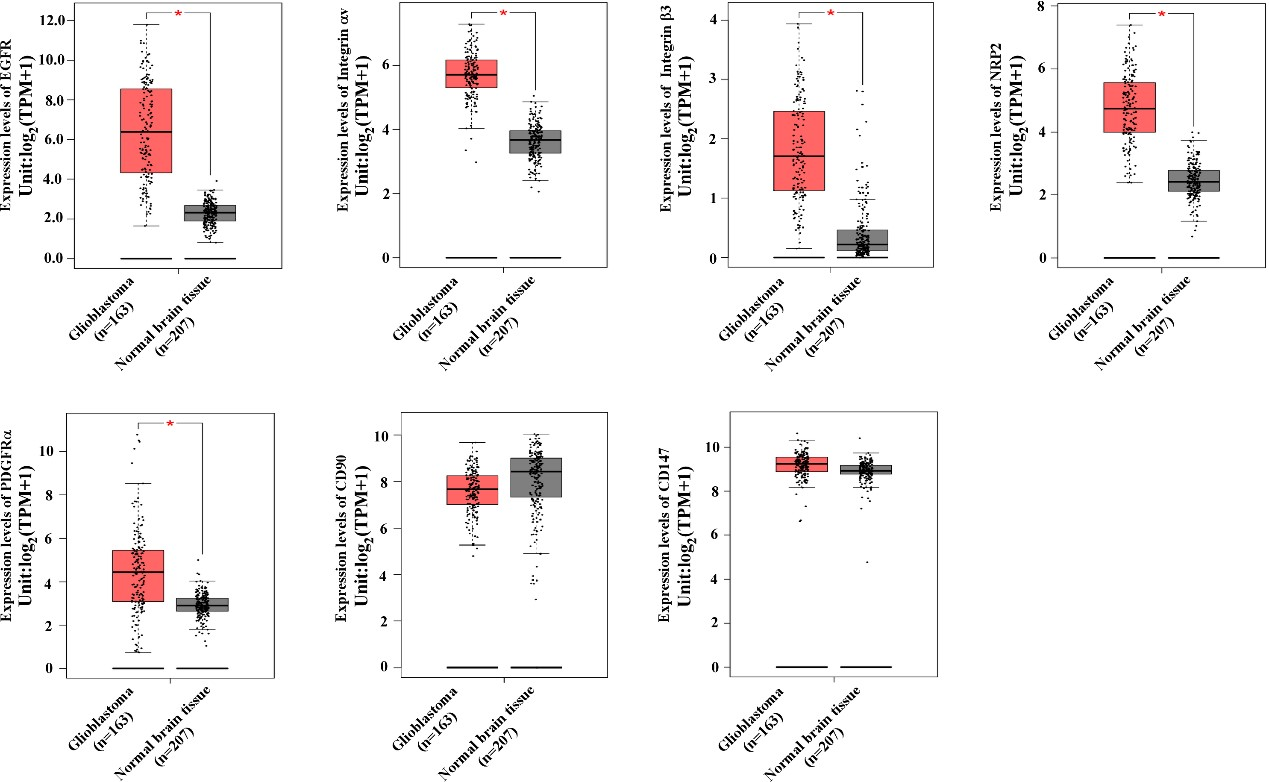

Supplement: S8 Fig — EGFR, Integrin αvβ3, Neuropilin-2, PDGFRα, CD90 or CD147 expression levels were analyzed in glioblastoma tissues and normal brain tissues using the GEPIA database. (TIF) [file ppat.1011304.s008.tif]
